# Supplementary material for: Enhanced Production and Functional Characterization of Recombinant Equine Chorionic Gonadotropin (rec-eCG) in CHO-DG44 Cells
Source: Biomolecules. 2025 Feb 14;15(2):289. doi: 10.3390/biom15020289 (PMC11853024; doi:10.3390/biom15020289)

Figure S1. Western blot analysis of rec-eCG proteins produced by monoclonal cells.

20201218

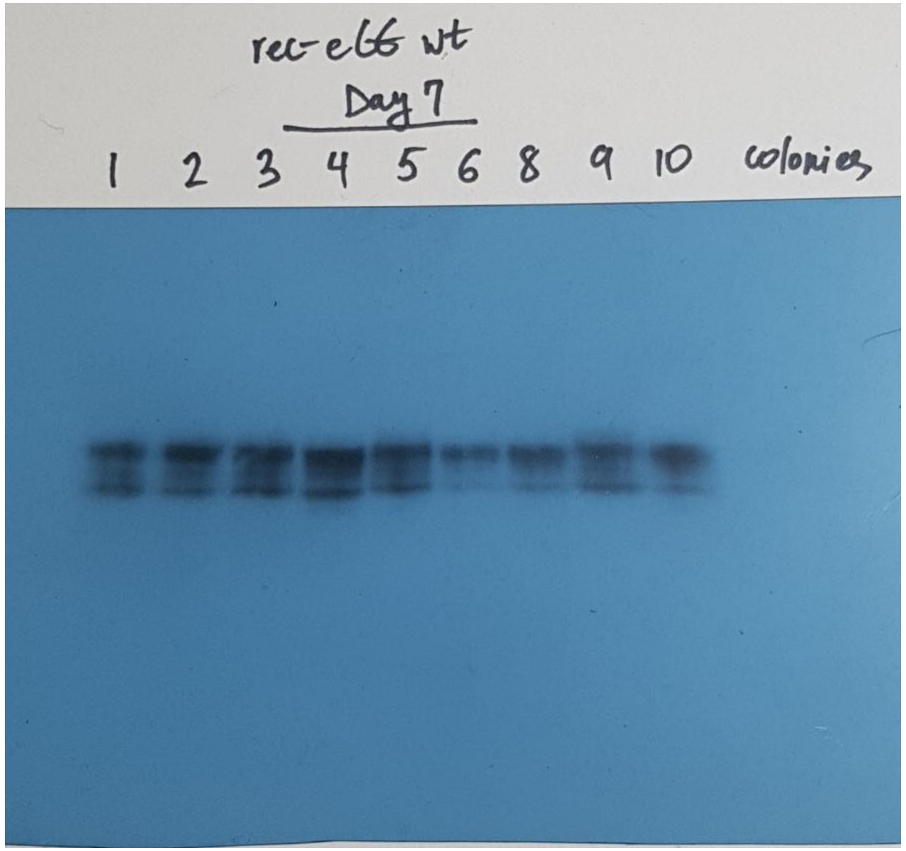

20201223

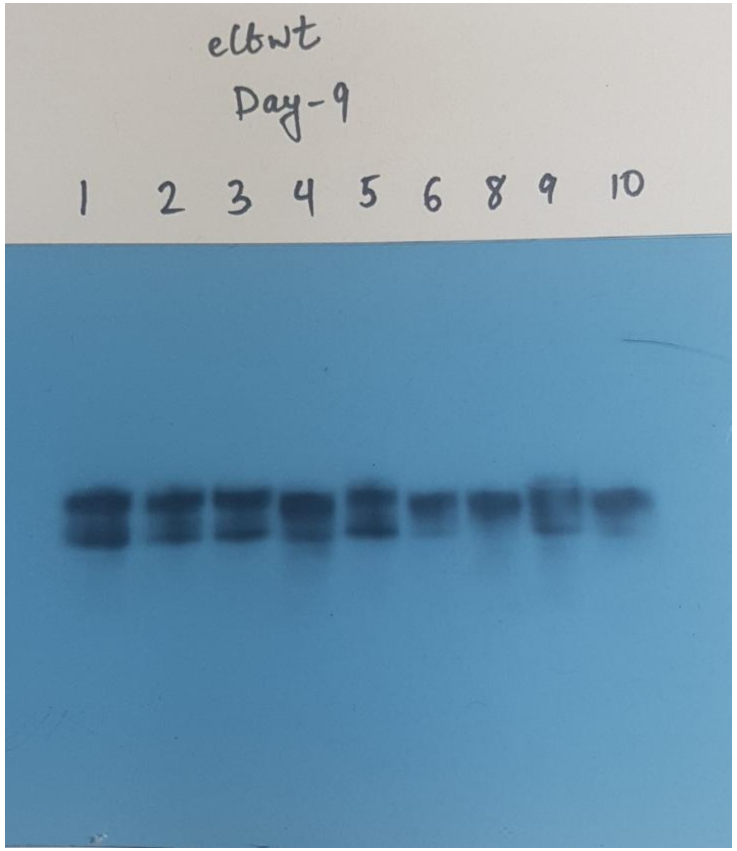

Figure S2. Western blot analysis of rec-eCG proteins over the cultivation period.

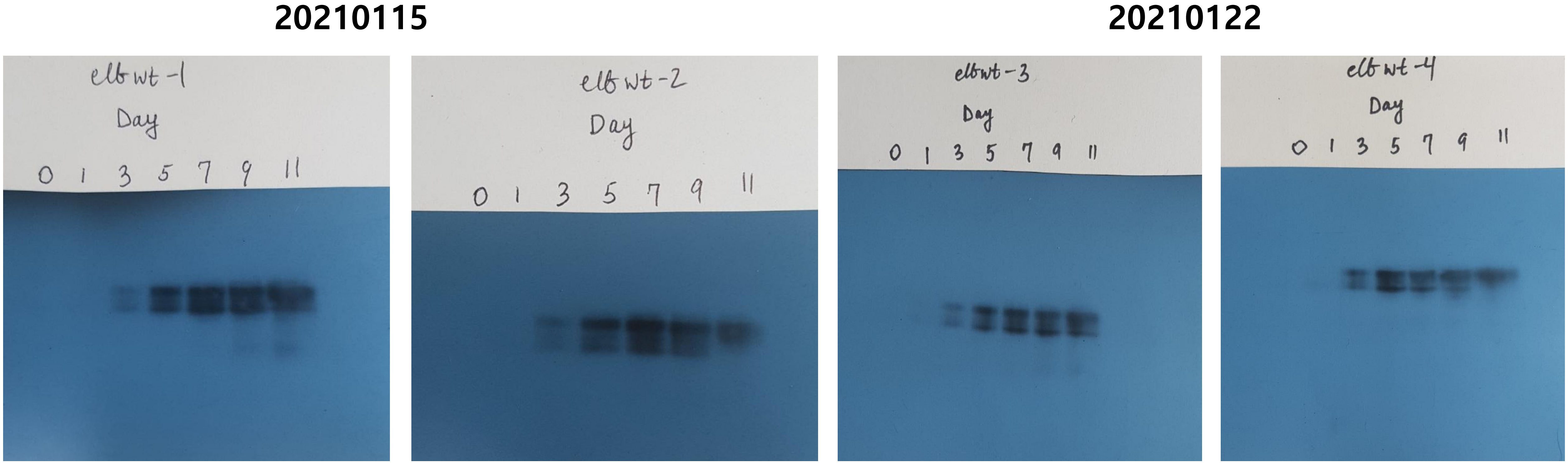

Figure S3. Deglycosylation analysis of rec-eCG proteins.

20210205

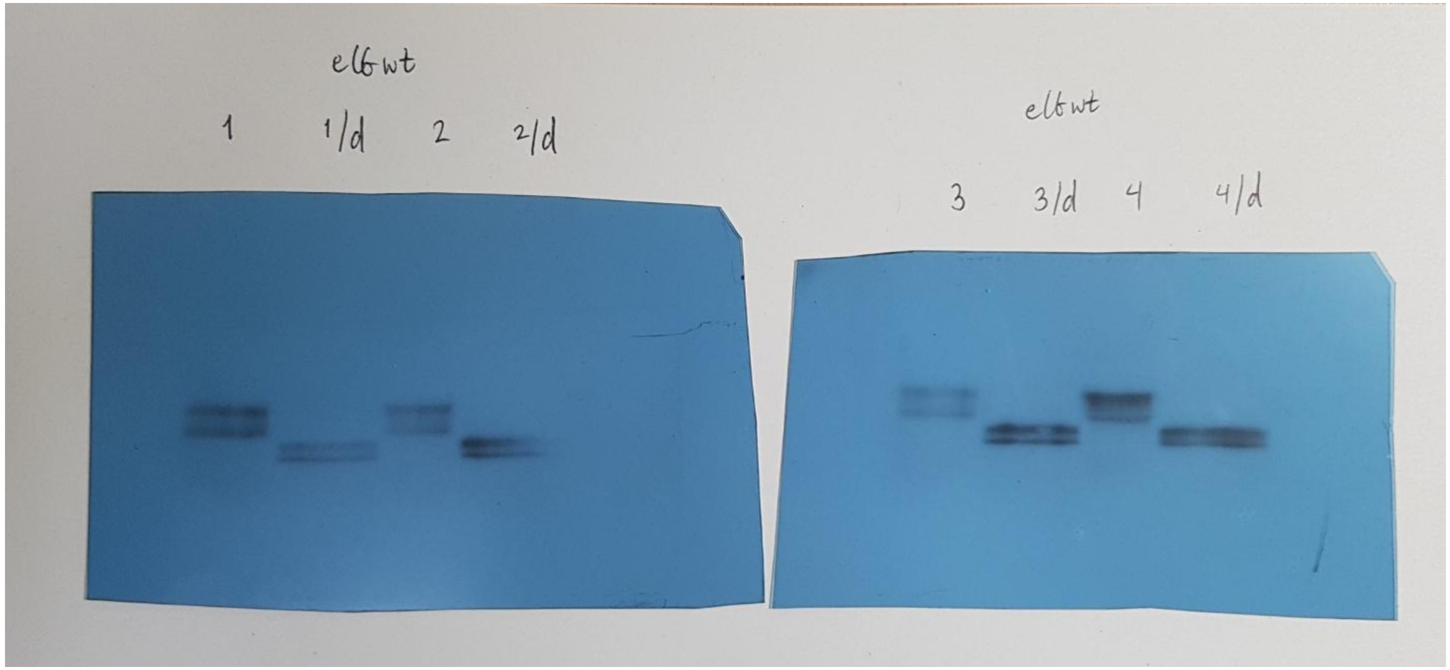

Figure S4. Effects of rec-eCG on pERK1/2 activation in eLH/CGR-stimulated cells.

A)

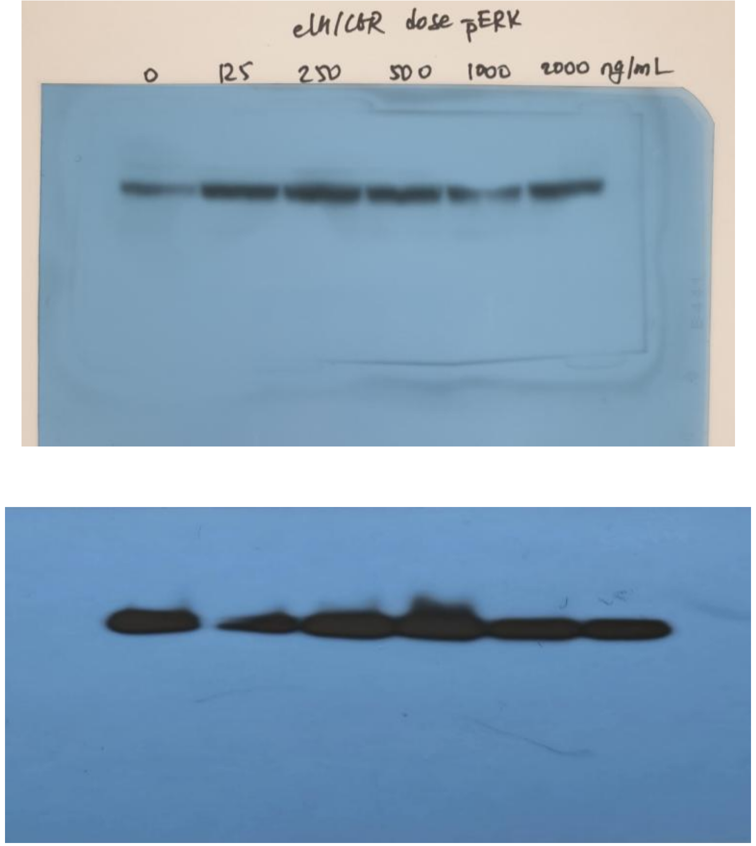

B)

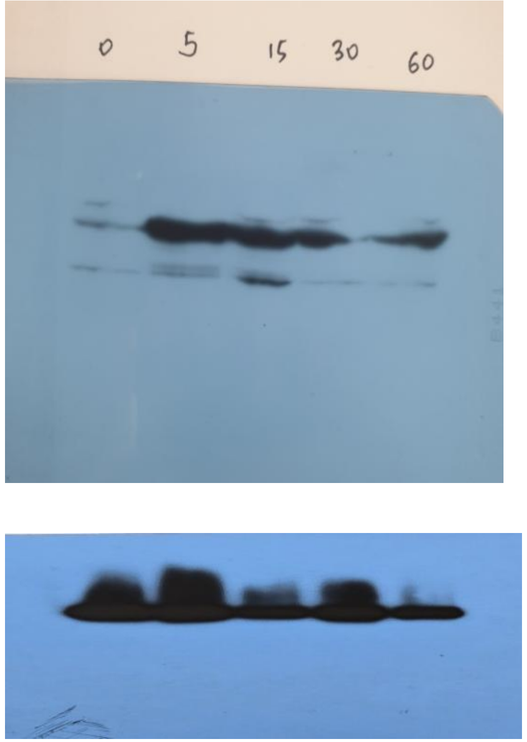

Figure S5. pERK1/2 activation stimulated by rLH/CGR and rFSHR

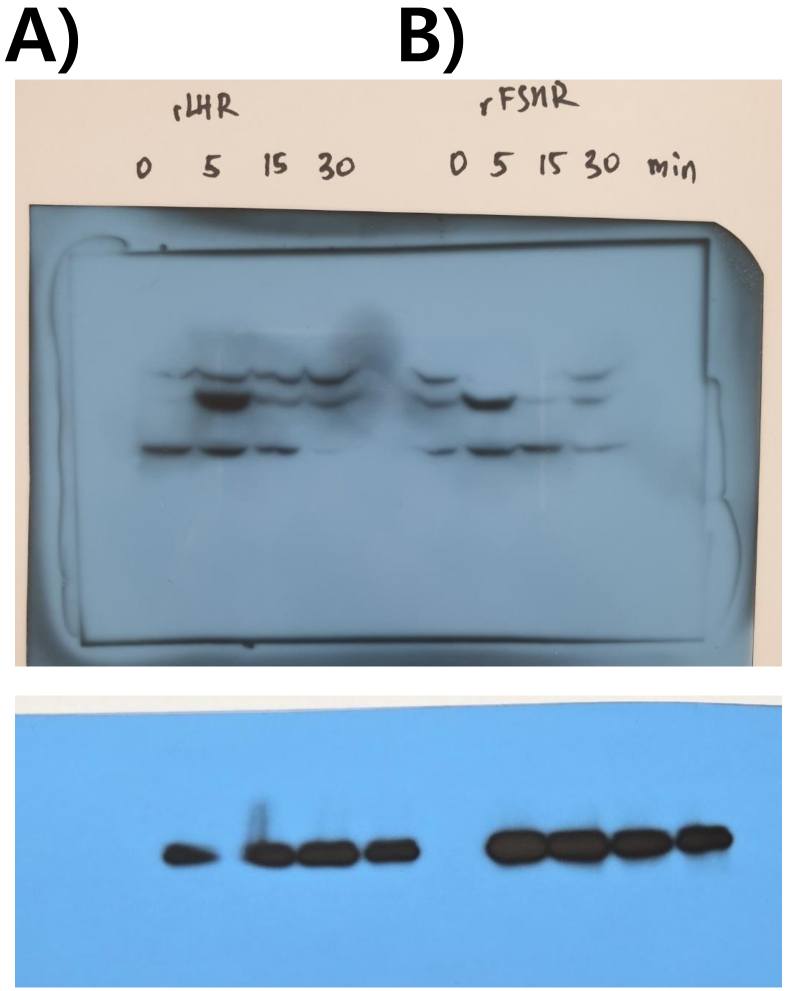

Supplement: Supplementary file 1 [file biomolecules-15-00289-s001.zip › biomolecules-3449994-supplementary.pdf]
